# Therapeutic sildenafil inhibits pulmonary damage induced by cigarette smoke exposure and bacterial inhalation in rats

**DOI:** 10.1080/13880209.2019.1711135

**Published:** 2020-01-22

**Authors:** Zhouxin Ren, Jiansheng Li, Junling Shen, Haibin Yu, Xiaofeng Mei, Peng Zhao, Zhenya Xiao, Wanliu Wu

**Affiliations:** a Academy of Chinese Medical Sciences, Henan University of Chinese Medicine, Zhengzhou, China; b Co-construction Collaborative Innovation Center for Chinese Medicine and Respiratory Diseases by Henan and Education Ministry of P. R. China, Zhengzhou, China; c First Affiliated Hospital of Henan University of Chinese Medicine, Zhengzhou, China

**Keywords:** Chronic obstructive pulmonary disease, pulmonary hypertension, therapeutic effect, emphysema, apoptosis of bronchiolar epithelial cell, pathological changes

## Abstract

**Context:**

Clinical reports showed sildenafil beneficial therapy on severe chronic obstructive pulmonary disease (COPD) with pulmonary hypertension (PH) patients.

**Objective:**

The study investigated therapeutic effects of silenafil on pulmonary damage induced by cigarette smoke exposure and bacterial inhalation in rats.

**Materials and methods:**

Female Sprague-Dawley rats (200–250 g) were divided into control group (no exposure, *n* = 10) and exposure group (*n* = 50) suffered from cigarette smoke exposure and *Klebsiella pneumonia* inhalation for 8 weeks. Then rats were orally given normal saline (control group or model group), 2.0, 3.0, or 4.5 mg/kg sildenafil for 4 weeks, respectively. Pulmonary pressure, RVHI and morphological analysis of pulmonary vascular remodeling, respiratory functions assay, morphological analysis of pulmonary alveoli, and expression of PCNA and caspase-3 of epithelial cells in bronchioles wall were examined.

**Results:**

Compared to model rats, 2.0, 3.0, and 4.5 mg/kg sildenafil increased VT by –0.6 to 9.58%, PEF by 3.12 to 6.49%, EF50 by 0.81 to 6.50%, decreased mPAP by 4.43 to 25.58%, RVHI by 6.54 to 26.41%, showing a dose-dependent improvement. Furthermore, 4.5 mg/kg sildenafil significantly increased MAN by 39.70%, LA/CSA by 37.07%, decreased muscular pulmonary arteries by 48.00%, WT by 12.83%, MT by 22.89%, caspase-3 expression by 17.71%, and showed improvement on abnormality in lung interstitial and bronchioles by microscopy.

**Discussion and conclusion:**

Our results demonstrated that sildenafil decreased pathological changes in alveoli, bronchioles, interstitial tissue, and arterioles of rats with COPD and PH.

## Introduction

Sildenafil is the first oral drug for pulmonary hypertension (PH) treatment by FDA recommendation. It is well-known that sildenafil is an efficient inhibitor of phosphodiesterase (PDE)5 which metabolizes the secondary messenger cyclic guanosine monophosphate (cGMP). Through inhibition of PDE5 by the drug, prolonged duration of cGMP exerts vasodilator and anti-proliferative actions to vascular smooth muscle cells in lung arterials, resulting in decrease of pulmonary arterial pressure and repression of vascular remodeling (Peinado et al. 1998; Barberà et al. 2001; Schermuly et al. 2008; Klinger et al. 2013). Besides its beneficial effect on pulmonary vessels, some reports showed sildenafil had some advantageous effect on chronic obstructive pulmonary disease (COPD) and PH patients (Barnes et al. 2019), such as increase of exercise capacity (Chen et al. 2015; Shrestha et al. 2017), improvement in the BODE index and quality of life (Vitulo et al. 2017), etc., suggesting possible inhibitory effect on lung abnormality.

Thus, animal COPD models were used for exploration of sildenafil on pulmonary damage. For guinea-pigs or mice, sildenafil reduced significantly airspace enlargement induced by cigarette smoke (CS), suggesting protective effect on emphysema in COPD patients (Domínguez-Fandos et al. 2015; Seimetz et al. 2015). Further study showed that cGMP played a key role in preserving lung structures through mediators such as vascular endothelial growth factor A and fibroblast growth factor 10, and antioxidant enzymes, such as superoxide dismutase, and anti-inflammation by preventing the activation and adherence of circulating inflammatory cells (Weissmann et al. 2014). However, in the above studies, duration of sildenafil administration was the same as the duration of the toxic substance attack, representing a preventive experimental design, in contrast to clinical research in which silde-nafil administration started and ended in the stage of COPD and PH, showing therapeutic design of sildenafil administration. Therefore, these experimental results could not accurately illustrate sildenafil therapeutic actions on pulmonary damage to COPD and PH.

In previous studies, a rat model induced by CS exposure and bacterial inhalation showed stable pulmonary damage and arterial remodeling, including of increase of bronchiole wall thickness, alveolar size, bronchiole stenosis and arteriole wall thickness, even in 20 weeks after stop of CS exposure and bacterial inhalation (Li et al. 2012). Therefore, the rat model was chosen for this study.

This work examined the therapeutic effect of sildenafil on pulmonary damage in COPD and PH induced by CS exposure and *Klebsiella pneumonia* inhalation in rats.

## Materials and methods

All experimental protocols were approved by the Experimental Animal Care and Ethics Committees of Henan University of Chinese Medicine (DWLL2018030063, Zhengzhou, China). Analyses of results were performed in a blinded and randomised fashion.

### Exposure to smoke and inhalation of *Klebsiella pneumonia*

Female Sprague-Dawley rats (200–250 g) were purchased from Laboratory Animal Center of NanJing Qing Long Mountain, China. Rats were divided randomly into control group (no exposure, *n* = 10) and exposure group (*n* = 50). According to a previous study (Li et al. 2012), whole body of the rats in exposure group was exposed to cigarette (Hongqi Canal^®^ Filter tip cigarette, tobacco type, tar: 10 mg, nicotine content: 1.0 mg, carbon monoxide: 12 mg, Henan Tobacco Industry, Zhengzhou, China) smoke of 8 cigarettes per treatment, twice a day, during the first two weeks; then 15 cigarettes per treatment, three times a day, from week 3 to week 8. *Klebsiella pneumonia* (KP; strain No: 46114) was provided by the National Center For Medical Culture Collections (Beijing, China). Suspension (100 μL) of *Klebsiella pneumonia* (6 × 10^8^ CFU/mL) was dropped into nasal cavities in the rats for 5 days, from week 1 to week 8. Then, the exposure and inhalation were stopped and the rats were treated as follows.

### Drug administration

Ten rats died of exposure to smoke and inhalation of *Klebsiella pneumonia,* and the remaining rats were divided into four groups (model; 2 mg/kg sildenafil; 3 mg/kg sildenafil and 4.5 mg/kg sildenafil) in accordance with almost equal pulmonary function impairment among the groups. A rat was administrated orally normal saline (2 mL/d) in control or model groups or different dose of sildenafil according to its group for 4 weeks.

### Respiratory function tests

Unrestrained whole-body plethysmography (UWBP, Buxco Electronics, Troy, NY) was applied for respiratory function tests weekly as previously described (Li et al. 2012). Tidal volume (VT), peak expiratory flow (PEF) and 50% tidal volume expiratory flow (EF50) were recorded and analyzed.

### Detection of pulmonary pressure, evaluation of right ventricular hypertrophy, and preparation of microscopy

On the end day of administration, five rats in each group were randomly chosen for pulmonary arterial pressure (PAP) assay, including of mean pulmonary artery pressure (mPAP), pulmonary artery diastolic pressure (PADP), and pulmonary artery systolic pressure (PASP). The rats were anaesthetized by 3% pentobarbital sodium (40 mg/kg) and a catheter (Portex FineBore Polythene Tubing, 0.58 mm, Smiths Medical International Ltd., Keene, NH) was inserted into pulmonary artery through right jugular vein, then, PAP was measured according to a report (Deten et al. 2003). Then, the rats were euthanized with 80 mg/kg pentobarbital sodium (absolute lethal dose, i.p.) and the catheter was back to right ventricle. After the rats died and aorta abdominals were cut off, sterile saline and 4% paraformaldehyde were successively perfused through the aforementioned catheter until colorless paraformaldehyde flew out from aorta abdominals. Other rats suffered from the same performances except PAP assay.

Trachea was exposed and 4% paraformaldehyde poured slowly into lung by trachea, then, carefully cut off bones and tissues combined with the lung. The complete lungs were taken from and put into 4% paraformaldehyde. Then, hearts were removed and were divided into the left ventricle plus septum (LV + S) and the right ventricle (RV) for right ventricular hypertrophy index (RVHI) evaluation. The RVHI was calculated according to the formula:

RVHI=RV/(LV+S).

After 12 h 4% paraformaldehyde fixation, left lobes of lung were cut into small pieces, embedded by paraffin, prepared in 5 μm sections and then stained with hematoxylin-eosin (H&E), Victoria blue or Van Gieson (VG), respectively.

### Morphological analysis of pulmonary arteries

Lung slices of VG stain were applied for pathologic changes of pulmonary arteries. Pulmonary arterioles from 50 to 200 μm diameters were captured at a magnification of 400 times with an Olympus PM-10AD optical microscope and photographic system (Olympus Optical Co., Ltd., Japan). The average external diameter (*D*) and complete vascular area, average lumen diameter (*L*) and lumen area, and average medial wall thickness (*M*) between internal and external elastic lamina of muscular arteries were measured by Image Pro Plus 6.0 software (Media Cybernetics, Inc., USA). The results were calculated as follows:

$$\text{Average vascular wall thickness \% }(\mathrm{WT}\%)\;=\frac{\left(D-L\right)}{D}\times \;100$$

$$\text{Average medial wall thickness \% }(\mathrm{MT}\%)\;=\;\frac{2M}{D}\;\times \;100$$

$$\text{Average lumen area \% }(\mathrm{LA/CSA}\%)\;=\;\frac{\text{lumen area}}{\text{vascular area}}\times \;100$$

In addition, all vessels were classified into nonmuscular vessels (only with an elastic lamina), partially muscular vessels (with a partial elastic lamina and a complete elastic lamina) and muscular vessels (with two complete elastic lamia). Then each type vessels percent of total vessels was calculated.

### Morphological analysis of alveoli

Slices stained by HE were applied for mean linear intercept (MLI) and mean alveolar numbers (MAN). The parameters of MLI and MAN were measured with IPP6.0 software; then, MLI and MAN were calculated as previously described (Voswinckel et al. 2004; Alejandre-Alcazar et al. 2007; Seimetz et al. 2011).

### PCNA and caspase-3 expression of epithelial cells in bronchioles

Expression of proliferating cell nuclear antigen (PCNA) and caspase-3 of epithelial cells in bronchioles was measured by immunohistochemistry. Paraffin-embedded lung tissue was sliced into 5 μm thick, deparaffinized and blocked with 3% hydrogen peroxide solution for 10 min to eliminate endogenous peroxidase activity, then incubated in polyclonal anti-PCNA or anti-caspase-3 antibody solution (Boster Biological Technology Co., Ltd, China; 1:100 dilution) overnight at 4 °C, respectively. After being washed with phosphate buffer solution 3 times, the slices were incubated with horseradish peroxidase (HRP)-labeled anti-rat immunoglobulin G (Boster Biological Technology Co., Ltd, China) and counterstained with hematoxylin. In each slice, all bronchioles were separated out from their outlines at 400 times magnification, then reserved for analysis with IPP 6.0 software. The ratio of integral optical density (IOD) of epithelial cells in total area of epithelial cells were calculated.

### Statistical analysis

All values were shown as mean ± standard deviation (SD). Data were analyzed using one-way analysis of variance (ANOVA) followed by S-N-K test (equal variances assumed) or Dunnett’s post hoc test (equal variances not assumed). Statistical significance was set at *p <* 0.05. All statistical analyses were performed with Statistical Product and Service Solution (SPSS), version 16.0 (Chicago, IL).

## Results

### Inhibition of sildenafil treatment on pulmonary hypertension, pulmonary vascular remodeling, and right heart hypertrophy

**Figure 1.**
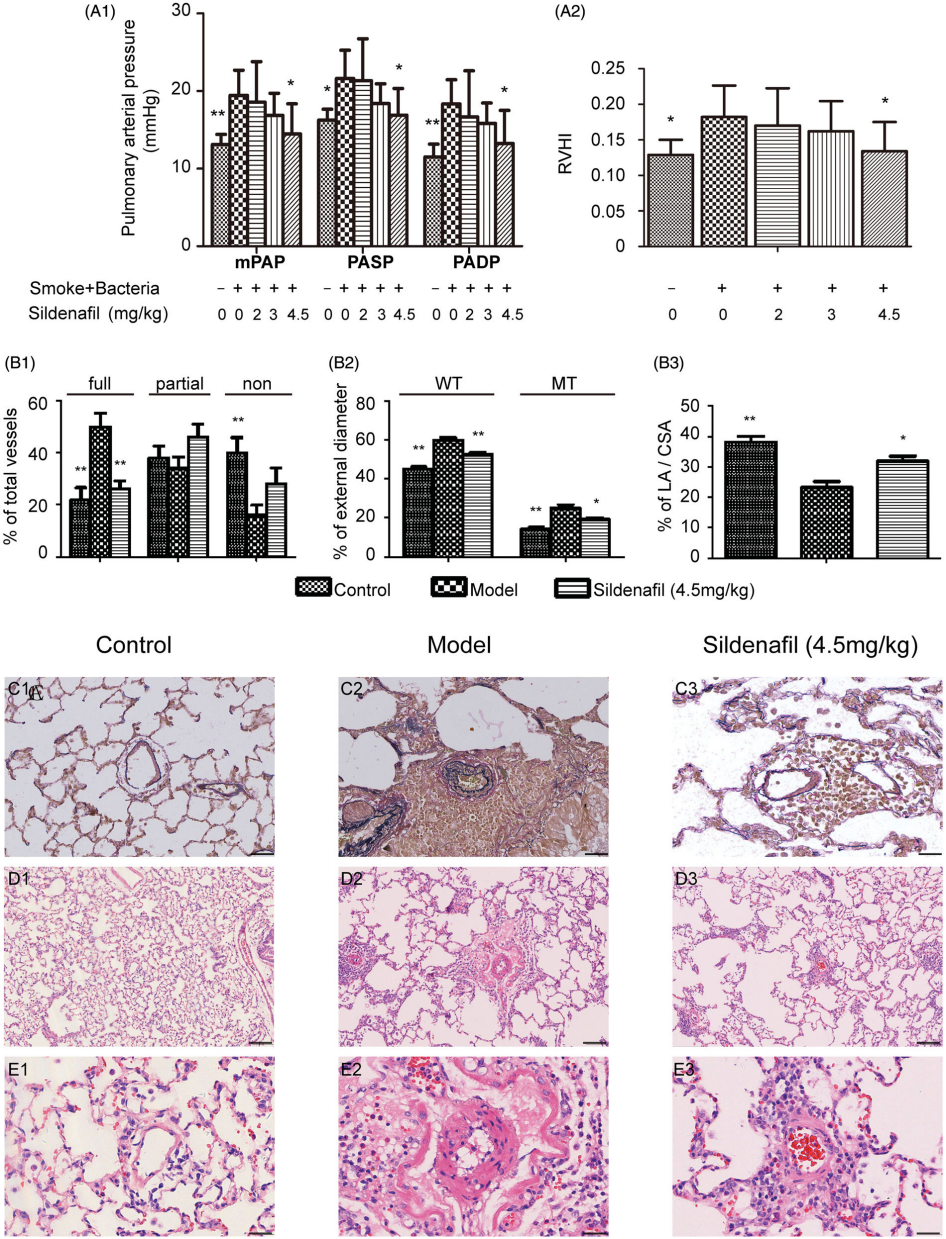
Effect of sildenafil on pulmonary hypertension, right heart hypertrophy and pulmonary vascular remodeling. Mean pulmonary artery pressure (mPAP), pulmonary artery systolic pressure (PASP) and pulmonary artery diastolic pressure (PADP) were shown in (A1). The ratio of right ventricular (RV) mass to left ventricular plus septum (LV + S) mass was shown in (A2). Percentage of nonmuscular (non), partially muscular (partial) and fully muscular (full) pulmonary arteries related to the total number of pulmonary arteries was shown in (B1). Percentage of vascular wall thickness (WT) and medial wall thickness (MT) related to vascular external diameter was shown in (B2). Percentage of lumen area (LA) related to complete vascular area (CSA) was shown in (B3). The above data were presented as mean ± SD; *n* = 10/group, except *n* = 5 in A1; *p < 0.05 vs. smoke and bacteria exposed group (Model group); **p < 0.01 vs. Model group. Representative photomicrographs of vascular remodeling stained by Victoria blue stain were shown in (C1, C2, and C3), with scale bars = 40 μm. Representative photomicrographs of vascular remodeling stained by hematoxylin and eosin stain were shown in (D1, D2, and D3), with scale bars = 160 μm, and were shown in (E1, E2, and E3), with scale bars = 40 μm.

Rats suffered from the exposure developed pulmonary hypertension (Figure 1(A1)) and pulmonary vascular remodeling which showed muscular vessels percent increase, nonmuscular vessels percent decrease, muscular vascular wall thickness increase and medial wall thickness increase, and vascular lumen stenosis (Figure 1(B1–B3)). Moreover, the rats also showed right ventricle hypertrophy (Figure 1(A2)). In comparison with model rats, 2.0, 3.0 and 4.5 mg/kg sildenafil decreased mPAP by 4.43, 13.28, and 25.58% (*p <* 0.05), PASP by 1.34, 14.93, and 21.88% (*p <* 0.05), PADP by 9.11, 13.58, and 27.77% (*p <* 0.05), RVHI by 6.54, 10.99, and 26.41% (*p <* 0.05), showing dose dependent inhibition (Figure 1(A1) and (A2)). Furthermore, in comparison with model rats, 4.5 mg/kg sildenafil decreased muscular pulmonary arteries by 48.00% (*p <* 0.01), WT by 12.83% (*p <* 0.01), MT by 22.89% (*p <* 0.05), increased nonmuscular pulmonary arteries by 75%, and LA/CSA by 37.07% (*p <* 0.05), showing that sildenafil inhibited vascular remodeling in several ways, including of inhibiting thickness increase of vascular walls and vascular media, and inhibiting vascular transforming from nonmuscular vessels to muscular vessels (Figure 1(B1–E3)).

**Figure 2.**
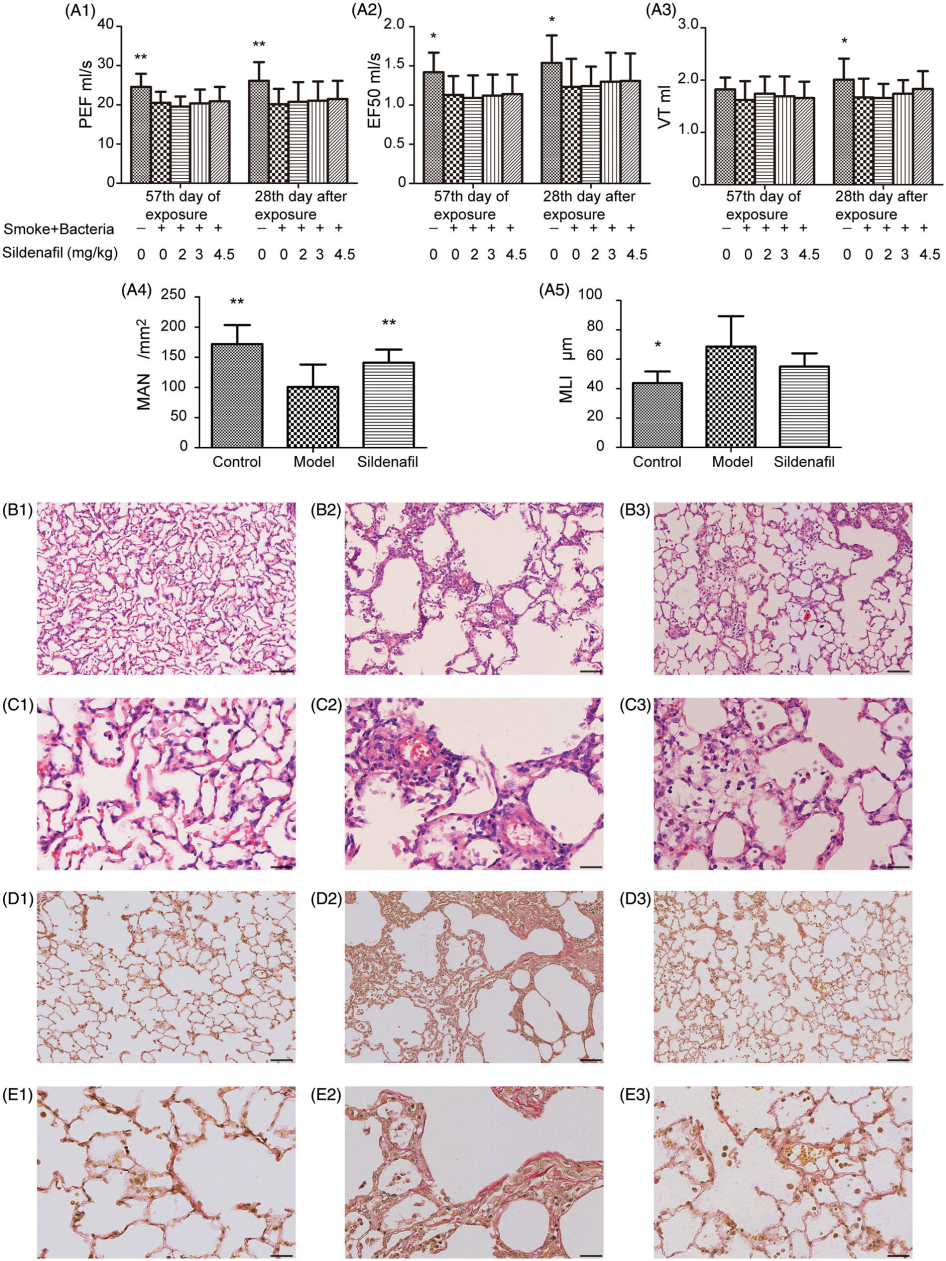
Effect of sildenafil on lung function, emphysema development and lung interstitial abnormality. Lung function quantified by peak expiratory flow (PEF), 50% tidal volume expiratory flow (EF50) and tidal volume (VT) was shown in (A1), (A2), and (A3). Emphysema evaluated by mean linear intercept (MLI) and mean alveolar numbers (MAN) was shown in (A4) and (A5). The above data were presented as mean ± SD; *n* = 10/group; *p < 0.05 vs. model group); **p < 0.01 vs. model group. Representative photomicrographs of emphysema development stained by hematoxylin and eosin stain were shown in (B1, B2, and B3) with scale bars = 160 μm, and in (C1, C2, and C3), with scale bars = 40 μm. Representative photomicrographs of interstitial abnormality stained by Van Gieson stain were shown in (D1, D2, and D3) with scale bars = 160 μm and in (E1, E2, and E3) with scale bars = 40 μm.

**Figure 3.**
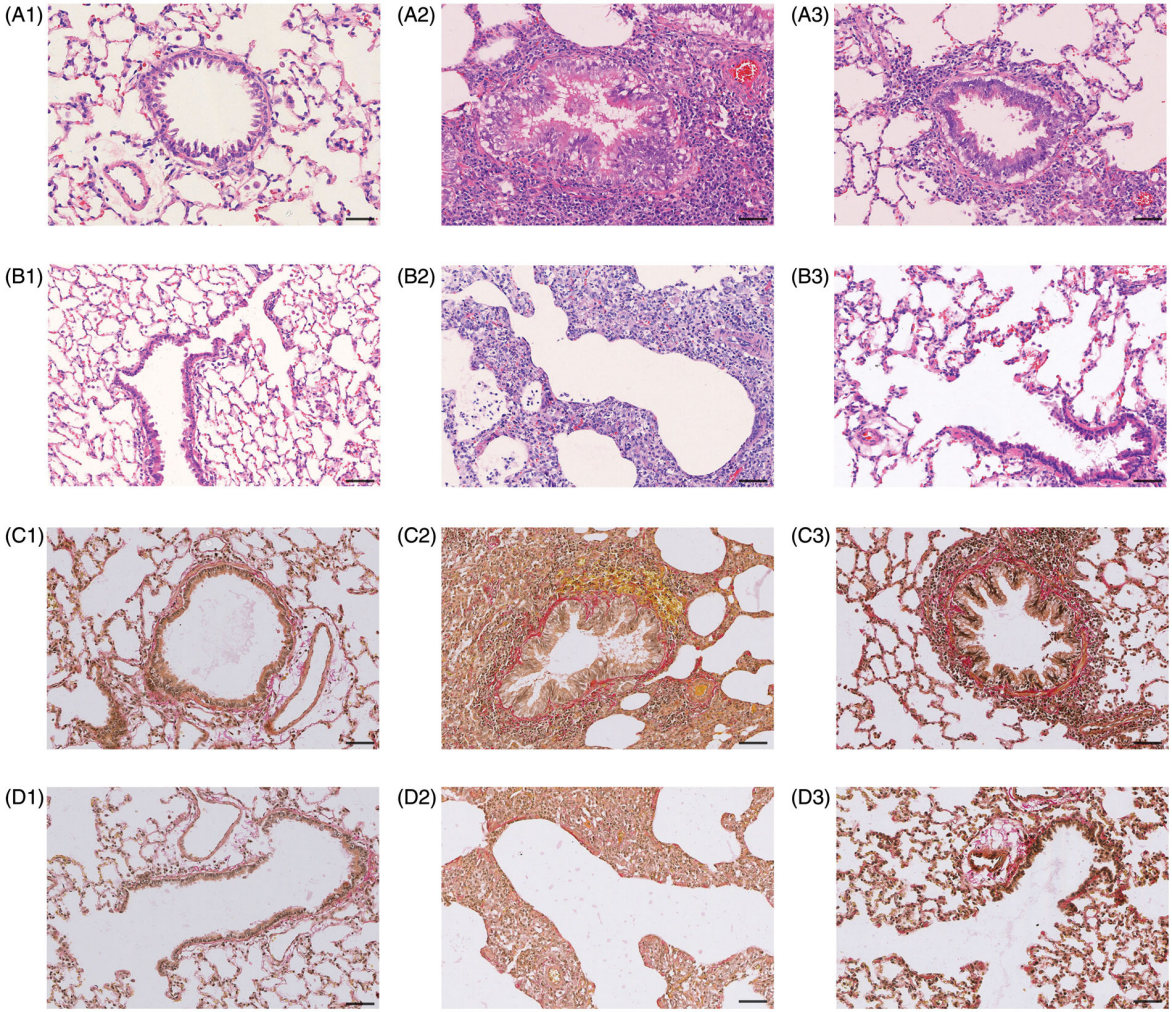
Effect of sildenafil on structural abnormality in bronchioles. Representative photomicrographs of bronchial abnormality stained by hematoxylin and eosin stain were shown in (A1, A2, and A3) with scale bars = 160 μm, and in (B1, B2, and B3), with scale bars = 40 μm. Representative photomicrographs of bronchial abnormality stained by Van Gieson stain were shown in (C1, C2, and C3) with scale bars = 160 μm, and in (D1, D2, and D3), with scale bars = 40 μm.

### Effect of sildenafil on pulmonary function

We examined three parameters of pulmonary function, VT, PEF and EF50, at 0 and 4 weeks after stop of smoke and bacteria exposure. Similar to previous studies (Li et al. 2012), pulmonary function decline in rats suffered from exposure sustained at least 4 weeks after exposure stop (Figure 2). In comparison with rats of model group, 2.0, 3.0 and 4.5 mg/kg sildenafil increased VT by –0.60, 4.19 and 9.58%, PEF by 3.12, 4.41, and 6.49%, EF50 by 0.81, 5.69 and 6.50%, with dose-dependent improvement (Figure 2). The results were consistent with a previous report, which showed that a similar PDE5 inhibitor, Tadalafil, could decrease pulmonary functional deterioration in COPD mice induced by tobacco smoke (Seimetz et al. 2015). However, the improvement might be weak due to no significant difference between a sildenafil group and model group (Figure 1). Furthermore, the results were insufficient for supporting for therapeutic action of sildenafil on pulmonary damage, therefore, other examinations were necessarily carried out.

### Effect of sildenafil on pathological changes of pulmonary tissue

In the study, model rats showed emphysema supported by MAN and MLI results (Figure 2(A4,A5)), and view of photomicrographs (Figure 2(B1–E3)), as similar as a report on mice COPD induced by tobacco smoke exposure (Seimetz et al. 2015). However, in the study, obvious increase in septal wall thickness could be viewed, with severe inflammatory cells infiltration in septal wall and interstitium, around broncholi and vessels (Figure 2(B1–C3)) in HE stains. Furthermore, VG stains showed obvious collagen fibrils accumulation in pulmonary interstitium. The above results suggested more severe pulmonary tissue damage in the rats than in the reported COPD mice, which might not cause a significant improvement of silden on pulmonary function. Sildenafil increased significantly MAN by 39.70% (*p* < 0.05) and decreased MLI by 19.82% (Figure 2(A4,A5)), suggesting inhibition for emphysema, as similar to the previous reports (Domínguez-Fandos et al. 2015; Seimetz et al. 2015). Furthermore, view of photomicrographs showed inflammation cells infiltration reduction in the sildenafil group rats as similar as a previous report (Seimetz et al. 2015), which might illustrate decrease of collagen fibrils accumulation in interstitium.

**Figure 4.**
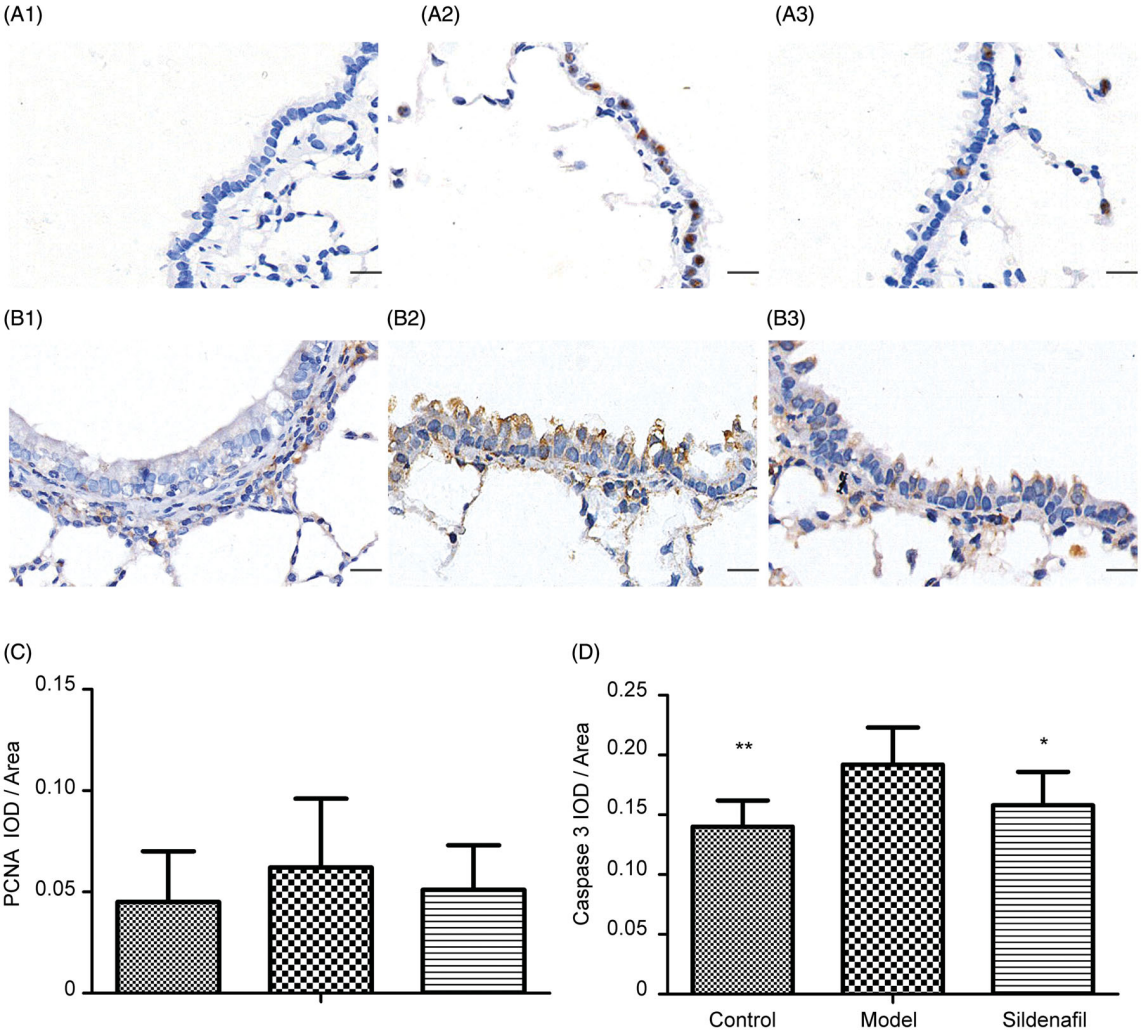
Effect of sildenafil on proliferating cell nuclear antigen (PCNA) and caspase-3 expression of epithelial cells in bronchioles. Representative photomicrographs of PCNA and caspase-3 expression in bronchioles with immunohistochemical stain were shown in (A1, A2, and A3 as PCNA) and (B1, B2, and B3 as caspase-3) with scale bars = 40 μm. The data of PCNA IOD/area was shown in (C) and the data of caspase-3 IOD/area was shown in (D). Data were presented as mean ± SD; *n* = 10/group; *p < 0.05 vs. model group); **p < 0.01 vs. model group.

### Effect of sildinafil on PCNA and caspase-3 expression of epithelial cells in bronchioles

Interestingly, HE and VG stains showed not only mucous epithelium hyperplasia, goblet cells proliferation and mucous increase in lumen (Figure 3(A2,C2)) but also cilia cells decrease and mucous epithelium exfoliation (Figure 3(B2,D2)), suggesting complex mixture of proliferation and apoptosis in epithelial cells of bronchioles of model rats. For further confirming the judgment, we examined the expression of two key proteins of bronchioles. One was PCNA which was often assessed cellular proliferation and another was caspase-3 which was often assessed apoptosis. In comparison to control group, PCNA and caspase-3 expression of model group increased by 37.78 and 32.91% (*p <* 0.01) (Figure 4(A1–D)), supporting coexistence of cellular proliferation and apoptosis in bronchiolar epithelial cells. Comparing to model group, 4.5 mg/kg sildinafil decreased PCNA by 17.74% and caspase-3 by 17.71% (*p <* 0.05) (Figure 4(A1–D)), appearing to significantly improve apoptosis of epithelial cells on bronchioles.

## Discussion

Previous studies have shown PAP reduction of sildenafil on experimental PH models induced by hypoxia, CS exposure and momocrotaline (Sebkhi et al. 2003; Schermuly et al. 2004; Weissmann et al. 2007; Domínguez-Fandos et al. 2015; Seimetz et al. 2015). In spite of discrepant results of pulmonary vascular remodeling, most of the studies supported anti-remodeling action of sildenafil (Sebkhi et al. 2003; Schermuly et al. 2004; Weissmann et al. 2007; Domínguez-Fandos et al. 2015; Seimetz et al. 2015). In the present study, sildenafil appeared to mediate a significant repression of PAP and vascular remodeling in rats. Further, the study illustrated sildenafil functioned in two ways to repress smooth muscle cell (SMCs) proliferation: one is reduction from nonmuscular vessels to muscular vessels; another is inhibitory thickness increase to the vascular media membrane. Moreover, similar to a previous study, the study showed reduction of lumen stenosis and right heart hypertrophy after sildenafil treatment (Schermuly et al. 2004).

Some clinical reports pointed out beneficial effects of sildenafil therapy on severe COPD with PH patients (Chapman et al. 2009; Blanco et al. 2010, 2013; Lederer et al. 2012; Rafiei et al. 2012; Sharif-Kashani et al. 2014; Chen et al. 2015; Shrestha et al. 2017; Vitulo et al. 2017). For further exploration of the mechanism leading to the above results, a severe COPD with PH animal model is essential. The model should appear not only pulmonary functional reduction and pulmonary pathological changes but also PH and pulmonary vascular remodeling. Although some studies reported stable pulmonary functional reduction and pulmonary pathological changes induced by CS exposure and *Klebsiella pneumonia* inhalation in rats (Li et al. 2012; Zhao et al. 2018; Ma et al. 2019), research of PH and pulmonary vascular remodeling in the rat model was deficient. In the study, besides of lung function drop and severe pulmonary lesions including of emphysema, inflammatory cells inflation and fibrosis accumulation increase in interstitium, model rats appeared to PAP increase and pulmonary vascular remodeling including of thickness increase of vascular walls and vascular media, and muscular vessels increase. Therefore, the rat model was suitable for valuation of therapy of drugs to COPD and PH. Furthermore, sildenal therapeutic administration but not preventive administration was more suitable to illustrate the results from clinical reports. However, in the previous studies (Sebkhi et al. 2003; Schermuly et al. 2004; Weissmann et al. 2007; Domínguez-Fandos et al. 2015; Seimetz et al. 2015), protective administration of sildenafil was carried out from exposure start to end.

In contrast to these studies, therapeutic administration of sildenafil was carried out from starting of COPD condition with drop of lung function; hence, the study gave more advantageous information. Sildenafil therapy did not significantly improve lung function and was different from previous reports, but showed lung function increase with dose dependence from 2.0 to 4.5 mg/kg. We inferred that a cause of the different results was more severe damage of lung induced by CS exposure and *Klebsiella pneumonia* inhalation in rats due to fibrosis accumulation increase in interstitium apart from emphysema in previous reports (Kiss et al. 2014; Weissmann et al. 2014; Domínguez-Fandos et al. 2015), and the another cause was low dose of sildenafil. Furthermore, therapeutic sildenafil could partly inhibit pulmonary lesions in the rats, such as decreasing emphysema supported by MAN result as similar as a previous report (Domínguez-Fandos et al. 2015), reducing interstitium fibrosis and inflammatory inflation. The results suggested anti-inflammation should be a main mechanism of sildenafil therapy, in line with some previous protective studies showing that sildenafil reduced neutrophil and mononuclear cells recruitment, decreased multiple cytokines and chemokines expression, and decreased inflammatory cell inflation (Kiss et al. 2014; Weissmann et al. 2014).

Furthermore, proliferation of epithelial cells, decrease of ciliated cells, exfoliation of epithelium, and infiltration of inflammation cells were observed in bronchioles of model rats, suggesting that damage was closely related to repair in epithelium of bronchioles. The regeneration and repair of this epithelium is a key process to ameliorate function and integrity of the small airways (Hogg et al. 2004; Beers and Morrisey 2011). Therefore, cell proliferation is a vital evaluation marker. PCNA is a nuclear protein and is expressed in the G1/M phase of the cell cycle. Due to close relation to DNA synthesis, the expression levels of PCNA is a common indicator for cell proliferation. In the study, in comparison with control rats, the expression in the epithelium of bronchioles showed no significant increase; in comparison with model rats, the expression showed no significant change in rats of 4.5 mg/kg sildenafil group. Furthermore, the evaluation of damage of epithelium in bronchioles was essential for sildenafil therapy. In the study, as a common indicator for apoptosis, caspase-3 expression in epithelium of bronchioles was assayed. Significant increase of its expression in model rats suggested aberrant increase of apoptosis, resulting in damage of epithelial cells in the bronchioles. In comparison with model rats, the expression in the sildenafil group was significant decreased, further supporting its therapeutic effect on pulmonary damage. For further unravelling therapeutic strategies of sildenafil, additional research will be essential for exploring processes of anti-apoptosis in the bronchiolar epithelium.

## Conclusions

The results indicated that therapeutic sildenafil reduced pulmonary damage induced by CS exposure and bacterial infection in rats, suggesting a close relation between anti-inflammation and inhibitory pulmonary damage. These findings contributed to a better understanding of sildenafil treatment for COPD and PH.
